# Supplementary material for: Flavones enrich rhizosphere Pseudomonas to enhance nitrogen utilization and secondary root growth in Populus
Source: Nat Commun. 2025 Feb 7;16:1461. doi: 10.1038/s41467-025-56226-w (PMC11805958; doi:10.1038/s41467-025-56226-w)
Supplement: Supplementary file 1 — Supplementary Information [file 41467_2025_56226_MOESM1_ESM.pdf]

Short Legends for Supporting Information

Supplementary Methods

Supplementary Methods 1. 16S rRNA gene sequencing and microbiome analysis.

Supplementary Methods 2. Root total RNA extraction, transcriptome sequencing and analysis.

Supplementary Methods 3. Metabolite measurement and metabolome analysis.

Supplementary Methods 4. Isolation and functional detection of pseudomonads.

Supplementary Methods 5. ^{15}N isotope dilution assay.

Supplementary Methods 6. DNA affinity purification sequencing (DAP-seq) and data analysis.

Supplementary Methods 7. Flavone quantification of root extract and root exudates.

Supplementary Figures

Supplementary Fig. 1 Phenotypic differences of nine species of poplar in four sections.

Supplementary Fig. 2 Root microbial community shaped by the vigorous genotype was more conducive to poplar growth.

Supplementary Fig. 3 Distinction of root microbiomes in poplar.

Supplementary Fig. 4 KEGG enrichment analyses of all differentially expressed genes.

Supplementary Fig. 5 Gene expression, flavonoid accumulation, and microbe enrichment pattern in poplar roots.

Supplementary Fig. 6 KEGG enrichment analyses of gene clusters.

Supplementary Fig. 7 Relative abundance of Pseudomonadaceae and *Pseudomonas* in the rhizosphere.

Supplementary Fig. 8 Determination of beneficial function of *Pseudomonas* strains.

Supplementary Fig. 9 Effect of pseudomonad inoculation on fitness in poplar.

Supplementary Fig. 10 Effect of pseudomonad inoculation on fitness in other plants.

Supplementary Fig. 11 *PopGL3* regulates flavonoid synthesis to shape the rhizosphere microbial composition of poplar.

Supplementary Fig. 12 Detection and identification of flavonoid metabolites signals in the root by LC-MS/MS.

Supplementary Fig. 13 Detection and identification of flavonoid metabolites signals in the root exudate by LC-MS/MS.

Supplementary Fig. 14 Abundance differences of rhizosphere microbiome between transgenic poplars and wild type.

Supplementary Fig. 15 Conceptual diagram of experimental design of this study.

Supplementary Fig. 16 Sterile hydroponic culture apparatus of poplar.

Supplementary Tables

Supplementary Table 1. Basic chemical properties of soils.

Supplementary Table 2. Contribution of biological N-fixation by different strains or SynComs to N nutrition of the shoots of poplars grown in soil containing ^{15}N.

Supplementary Table 3. Contribution of biological N-fixation by transgenic plant root microorganisms to N nutrition of the shoots of poplars grown in soil containing ^{15}N.

Supplementary Table 4. Details of nine poplar species in four sections.

Supplementary Table 5. Details of four poplar forest soils.

Supplementary Methods 1 16S rRNA gene sequencing and microbiome analysis

(1) 16S rRNA gene sequencing

The genomic DNA from the rhizosphere soil and bulk soil samples was extracted using the CTAB method. The 16S rRNA genes from specific regions (16S V3-V4) were amplified using specific primers (V3-V4: 341F-806R) that included barcodes. Each PCR reaction consisted of 15 μl of Phusion® High-Fidelity PCR Master Mix (New England Biolabs, USA), 0.2 μM of forward and reverse primers, and approximately 10 ng of template DNA. Subsequently, the PCR products were purified using the Gel Extraction Kit (Qiagen, Germany).

Sequencing libraries were prepared using the TruSeq® DNA PCR-Free Sample Preparation Kit (Illumina, USA) according to the manufacturer's instructions, and index codes were added. The quality of the libraries was assessed using the Qubit@2.0 Fluorometer (Thermo Scientific, USA) and the Agilent Bioanalyzer 2100 system (USA). Finally, the libraries were sequenced on an Illumina NovaSeq platform (USA), generating 250 bp paired-end reads.

(2) Amplicon data analysis

The 16S rRNA gene sequences were processed using QIIME[1] (v.1.9.1), USEARCH (v.10.0)[2] and in-house scripts. The quality of the paired-end Illumina reads was checked by FastQC[3] (v.0.11.5) and processed in the following steps by USEARCH: joining of paired-end reads and relabeling of sequencing names (-fastq_mergepairs); removal of barcodes and primers (-fastx_truncate); filtering of low-quality reads (-fastq_filter); and finding non-redundancy reads (-fastx_uniques). Reads were clustered at 100% sequence similarity using Unoise3 with default parameters[4]. Taxonomic assignment utilized the SILVA reference database (v132) for bacteria[5]. One table was created for each taxonomic level (Domain, Phylum, Class, Order, Family, and Genus)[6,7]. All absolute sequence variants (ASVs) identified as chloroplast and mitochondria were discarded from the dataset, and ASVs represented by fewer than two sequences were filtered to avoid biases. The QIIME (v.1.9.1) was selected to evaluate the alpha diversity index (Shannon index, Chao1 index) and beta diversity

index (weighted/unweighted Unifrac distance and Bray-Curtis dissimilarity) of microbial communities among all soil samples.

(3) Statistical analysis

Linear discriminate analysis effect size (LEfSe) was used to analyze the differentially taxonomical features among microbial communities from different sections[8]. Statistical analyses were based on the FDR-corrected Kruskal-Wallis test. The significant taxonomical biomarkers were selected with FDR adjusted P-value < 0.05 and logarithmic LDA score > 2. STAMP[9] (v.2.1.3) software was used to analyze the differences between overexpression plants (*PopCHS4-OE* and *PopGL3-OE*) and wild-type poplar rhizosphere microbial communities. Rhizosphere microbial differences among overexpression plants (*PopCHS4-OE* and *PopGL3-OE*) and wild-type were re-analyzed using ANCOM-BC2[10] to increase the reliability of the results.

Supplementary Methods 2 Root total RNA extraction, transcriptome sequencing and analysis

(1) Root total RNA extraction and transcriptome sequencing

Total RNA was extracted from the root tissues of nine poplar species. The frozen roots were fully pulverized using liquid nitrogen, and the RNA molecules were then meticulously extracted via the utilization of the RNeasy Mini Kit (Qiagen, Germany) following the instructions provided by the manufacturer. Rigorous scrutiny was applied to evaluate the quality of the extracted RNA through agarose gel electrophoresis, ensuring the presence of distinct, unambiguous bands. The RNA concentration and the 260/280 nm ratio were determined using a NanoDrop 2000 spectrophotometer (Thermo Scientific, USA). Samples meeting rigorous quality criteria were directed towards the construction of RNA-seq cDNA libraries. Subsequent sequencing procedures, carried out on the HiSeq 6000 platform (Illumina, USA), were performed by Novogene Co., Ltd., Tianjin, China, meticulously aligned

with the manufacturer's instructions. Finally, paired terminal reads of 150 bp were acquired.

(2) Transcriptome analysis

Transcriptome profiling was performed as described previously[11]. In brief, the initial raw RNA-seq data underwent a rigorous quality filtration process employing the fastp software[12] (v.0.14.0). Paired reads possessing adapters, exhibiting an N content < 10% or with > 50% low-quality nucleotides (sQ ≤ 5) were methodically eliminated from the dataset. Clean reads obtained from the Hiseq X Ten sequencing platform were mapped to the *P. trichocarpa* reference genome (v.3.1; https://phytozome-next.jgi.doe.gov/info/Ptrichocarpa_v3_1) using HISAT2[13] (v.2-2.1.0), the mapped sam files were converted to bam files and sorte using samtools (v.0.1.19) then normalized to TPM using StringTie[14] (TPM > 0; v.1.3.6). The PCA and HCA were performed as described above. Differentially expressed genes were analyzed using DESeq2 (v.1.34.0; $|\log2FC| \geq 1$, *FDR* adjusted *P*-values < 0.05). The functional enrichment analysis was analyzed by KOBAS[15] (v.3.0) software.

Supplementary Methods 3 Metabolite measurement and metabolome analysis

(1) Metabolites extraction

Fine-root samples (< 2 mm diameter; 100 mg) of nine poplar genotypes (three independent replicates; 27 samples in total) were meticulously grounded using liquid nitrogen, and the homogenate was reconstituted with prechilled 80% MeOH and 0.1% formic acid through a vortexing process. The concoctions were then centrifuged at 10,000 g and 4 °C for 20 min. A portion of this supernatant was diluted to a final concentration containing 53% MeOH. The processed samples were subsequently transferred to new centrifugal tubes and underwent another round of centrifugation at 10,000 g and 4 °C for 20 min. The resulting supernatants were introduced into the LC-MS/MS system for analysis. To ensure instrument stability, the samples were equally amalgamated to form multiple quality control samples (QC; four samples).

(2) HPLC-MS/MS analysis

LC-MS/MS analyses were performed using an ExionLC™ AD system (SCIEX, USA) coupled with a QTRAP® 6500+ mass spectrometer (SCIEX, USA). The samples were introduced onto a Xselect HSS T3 (2.1×150 mm, 2.5 μm) employing a 20-min linear gradient at a flow rate of 0.4 ml/min. The eluents utilized were eluent A (0.1% Formic acid-water) and eluent B (0.1% Formic acid-acetonitrile). The solvent gradient was designed as follows: 2% B, 2 min; 2-100% B, 15.0 min; 100% B, 17.0 min; 100-2% B, 17.1 min; 2% B, 20 min. The QTRAP® 6500+ mass spectrometer was set to operate in positive polarity mode with an IonSpray Voltage of 5500 V and a Temperature of 550 °C. Similarly, for the negative polarity mode, the QTRAP® 6500+ mass spectrometer was operated with an IonSpray Voltage of -4500 V, while maintaining the Temperature at 550 °C.

(3) Metabolites identification and quantification

The analysis of samples utilizing Multiple Reaction Monitoring (MRM) was based on Novogene's proprietary database. Metabolite quantification relies on Q3 for accurate measurements. The Q1, Q3, RT (retention time), DP (declustering potential), and CE (collision energy) were used for metabolite identification. The data files produced through HPLC-MS/MS were processed using SCIEX OS (v.1.4), facilitating peak integration and correction. The primary parameters were configured as follows: minimum peak height set at 500, signal-to-noise ratio at 5, and Gaussian smooth width established as 1. Importantly, the area encompassed by each peak accurately depicts the relative content of the corresponding substance.

(4) Metabolome analysis

Partial least squares discriminant analysis (PLS-DA) was executed using metaX[16] (v.2.71). For statistical significance, we employed univariate analysis based on two sided (t-test). Metabolites surpassing a VIP > 1 along with a P-value < 0.05 and exhibiting a $|\log2FC| \geq 1.585$ (i.e., fold change ≥ 3 or ≤ 0.333) were classified as differential metabolites.

Supplementary Methods 4 Isolation and functional detection of pseudomonads

(1) Isolation of pseudomonads

The poplar rhizosphere soils collected from the pots with the mixed soil samples cultivated with *Leuce* (Pto-M, 84K, Pal-Y, and LM50) were diluted using a 10-fold dilution method to a dilution of 10^{-5}. Subsequently, 200 μl of each 10^{-4} and 10^{-5} soil dilution were separately spread onto KB and LB agar plates, with three replicates for each gradient. The plates were then incubated at a constant temperature of 28 °C for 1-2 days. Colonies with distinct morphologies were selected for identification and preservation. The 16S rRNA genes (V3-V6) were cloned from all single strains using the specific primer (341F-1046R; Supplementary Data 12) and sequenced using the Sanger method.

(2) *nif*H gene cloning

The purified *Pseudomonas* strains were subjected to PCR amplification of the *nif*H gene using the nested approach, aiming to ensure the precision of the results. The target fragment length was approximately 360 bp. The first-round specific primers were FGPH19 and POLR, while the second-round primers were AQER and POLF (Supplementary Data 12). Gel electrophoresis was employed for detection, and the presence of bands within the range of 300–400 bp was preliminary evidence of nitrogen-fixing capability.

(3) Detection of the nitrogen fixation ability of the strains

The pseudomonad strains were cultivated in KB liquid medium to an OD600 of 1.0. Subsequently, 5 μl of the bacterial suspension was inoculated onto Ashby nitrogen-free solid medium. Each strain had three replicates. The cultures were maintained at a constant temperature of 28 °C for 4–7 days. Observe whether there are nearly transparent colonies.

(4) Detection of inorganic phosphorus hydrolysis ability in strains

The 5 μl of the aforementioned bacterial suspension was inoculated onto PKO inorganic phosphate solid medium; each strain had three replicates. The cultures were

incubated at a constant temperature of 28 °C for 4–7 days. The formation of clear zones around the colonies was observed as an indicator of phosphate solubilization.

(5) Detection of IAA secretion capacity in strains

The pseudomonad strains were cultivated in R2A medium, with the addition of 0.1% tryptophan, and incubated at 28 °C for 72 h. Standard curves for Indole-3-acetic acid (IAA) were established using concentrations of 0, 1, 5, 10, 30, and 50 µg/ml in R2A medium. The Salkowski reagent[17] was employed for the colorimetric assay, composed of 4.5 g $FeCl_3$, 22.3 ml H_2SO_4, and 77.7 ml water. A mixture of 1 ml Salkowski reagent and 1 ml sample solution was incubated in darkness for 30 min at 40 °C. The absorption spectra of the resulting 200 µl mixture were measured at 530 nm using 96-well plates. Each sample was subjected to three technical replicates.

Supplementary Methods 5 ^{15}N isotope dilution assay

To verify the nitrogen-fixing potential of *Pseudomonas* strains in poplar (84K) and assess the impact of transgenic plants on root microbiota nitrogen-fixing capacity, $(NH_4)_2SO_4$ labeled with ^{15}N (10% ^{15}N atom, MACKLIN, China) was applied as a substitute for nitrogen fertilizer to the transgenic plants or inoculated poplars with the above isolates (20 ml 1 g/L $(NH_4)_2SO_4$ per pot). After eight weeks, leaves from the 3rd, 4th, and 5th nodes from the top were collected for each treatment. The leaves were dried thoroughly at 60 °C and ground into a fine powder.

The nitrogen content and ^{15}N enrichment of the leaves were analyzed using the Elementar Vario PYRO Cube elemental analyzer (Vario PYRO Cube, Germany) and the Isoprime 100 isotope mass spectrometer (Isoprime, United Kingdom). The calculation of biological nitrogen fixation (BNF) takes the inoculation experiment of isolates as an example[18]:

$$\%Ndfa = (1 - \%^{15}Ndf\mathit{i}/\%^{15}Ndf\mathit{ai}) * 100$$

$$N_2\text{-fixed} = \%Ndfa * Ntotal$$

where %Ndfa represents the percentage of N derived from the air, %^{15}Ndf refers to the enrichment in plants inoculated with isolates (*i*) and autoclaved isolates (*ai*),

N_2-fixed represents N derived from the air, and Ntotal indicates the total N content of the entire inoculated plant. The transgenic plant roots, collected following the previously mentioned procedure, were stored at -80 °C for flavone quantification.

Supplementary Methods 6 DNA affinity purification sequencing (DAP-seq) and data analysis

The DAP-seq experiment was conducted by Beijing Huizhi Bio Ltd. following the procedures described earlier[19]. In brief, the cDNA sequence of *PopGL3* (Supplementary Data 13) was connected to the pFN19K vector using One-step cloning technology. Subsequently, the expression of the PopGL3 protein was induced using the TnT® Coupled Wheat Germ Extract System (Promega, USA). Genomic DNA (gDNA) was extracted from the fresh leaves of the 84K poplar. The gDNA was sonicated to create fragments with sizes ranging from 200 to 800 bp. The Halo-PopGL3 protein was bound to anti-Halo monoclonal antibody agarose beads (Promega, USA), and these beads were incubated with 200 ng of fragmented gDNA for 1 h at room temperature. After incubation, the beads were washed, and DNA was recovered. The samples were combined and subjected to sequencing on the Illumina Novaseq 6000 platform (USA), generating an average of 150 bp paired-end reads for each library. Each sample produced a total of 10–30 million reads. The analysis incorporated two biological replicates.

Sequencing reads were subjected to trimming using Trimmomatic, with the specified parameters: ILLUMINACLIP:TruSeq3-PE.fa:2:30:10:8:true LEADING:20 TRAILING:20 SLIDINGWINDOW:4:20 MINLEN:50. The trimmed reads were mapped to the 84K poplar genome (https://www.ncbi.nlm.nih.gov/datasets/genome/GCA_033617975.1/) using Bowtie2 software (v.2.3.5). Mapped reads were filtered to retain uniquely mapped reads using SAMtools software. These uniquely mapped reads were employed for all subsequent analyses. MACS2 software was used for peak calling. The association of DAP-seq peaks located within 2 kb upstream or downstream of the transcription start site (TSS)

was analyzed using BEDtools, following the General Feature Format (GFF) files. The coverage of peaks across chromosomes and profiles of peak bindings to TSS regions were analyzed using ChIPseeker software (v.1.22.1). Motif discovery was conducted using Homer software (v.4.11).

Supplementary Methods 7 Flavone quantification of root extract and root exudates

Following the hydroponic cultivation method described above, both transgenic and wild-type poplars were grown in a sterile, equal-volume sucrose-free 1/2 MS solution, with three independent replicates for each genotype (a total of nine samples). After 20 days, root exudates were collected and passed through a 0.2-μm filter. Subsequently, 10 ml of root exudate was freeze-dried and then resuspended in 250 μl of 50% MeOH containing 0.1% formic acid. For root extracts, 0.5 g fresh root samples (three independent replicates; a total of nine samples) of transgenic and wild-type poplars collected in the above ^{15}N isotope dilution assay were ground into fine powder in liquid nitrogen, dissolved in 99% MeOH, freeze-dried, and resuspended in 250 μl of 50% MeOH with 0.1% formic acid. Target metabolites (tricin, apigenin, and luteolin) were analyzed on an Agilent 1290 lnfinity-6470 triple quadrupole mass spectrometer (USA) equipped with a Gemini C18 column (250×4.6 mm, 5 μm). The mobile phase consisted of (A) 0.1% acetic acid and (B) acetonitrile, which was run at a flow rate of 0.2 ml/min. The gradient was programmed as follows: 10% B for 5 min, increased to 100% at 15 min, and the column temperature was set at 30 °C. The concentration of each compound was calculated by normalizing the peak area to that of the standard curve of its corresponding analytical standard.

Supplementary Figures

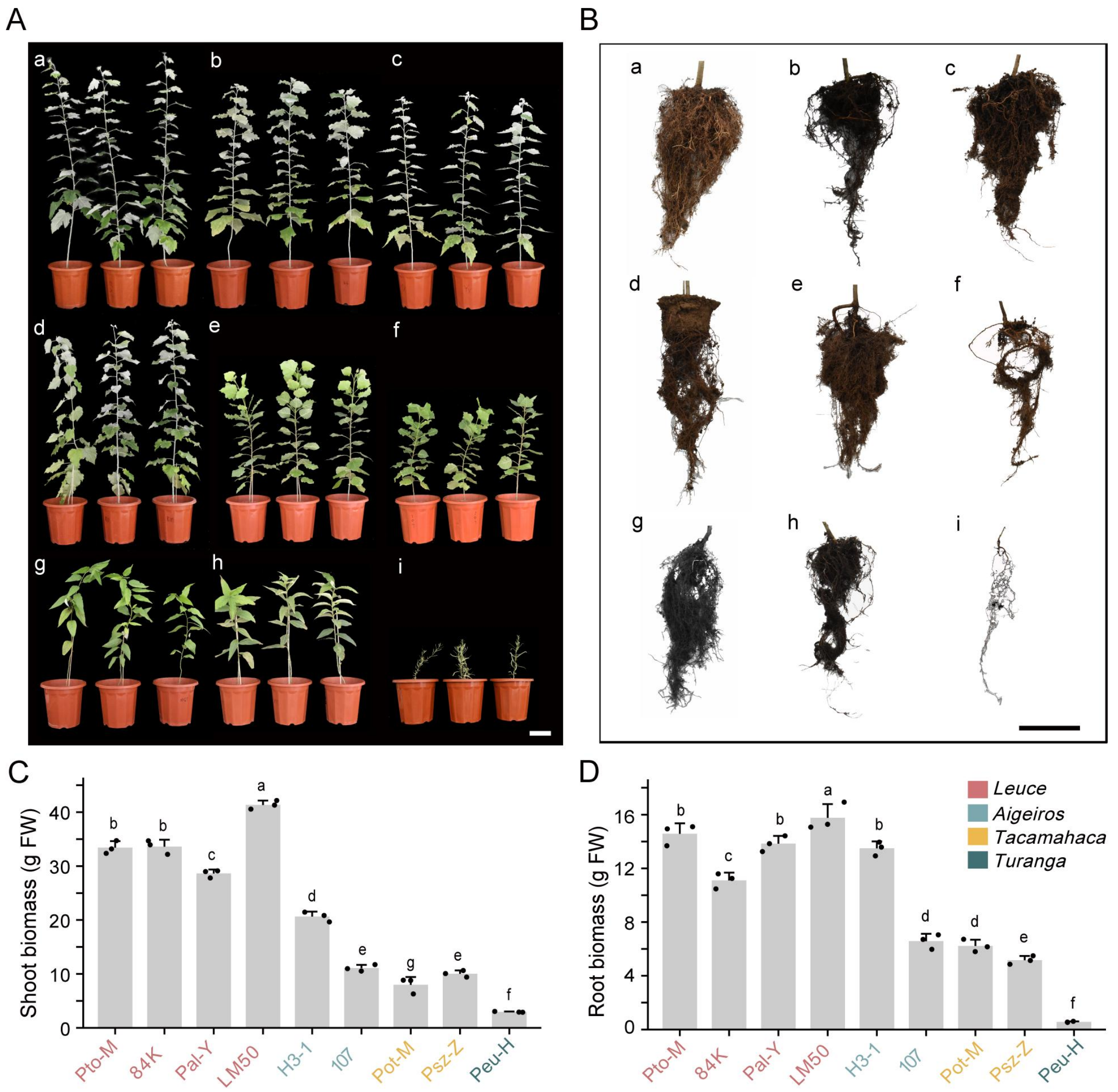

Supplementary Fig. 1 Phenotypic differences of nine species of poplar in four sections. Growth differences of the whole plant **(A)** and root **(B)** between nine poplar species growing in natural soil mixture. a: Pto-M; b: 84K; c: Pal-Y; d: LM50; e: H3-1; f: 107; g: Pot-M; h: Psz-Z; i: Peu-H. Fresh shoot **(C)** and root **(D)** biomass of nine poplar species growing in natural soil mixture. Species of the same color belong to the same section. *n* = 3 biologically independent samples. Different letters indicate significantly different groups (One-way ANOVA, *P*-values < 0.05; *P*-values are shown in Source Data file). Each bar represents the mean ± SEM. FW, fresh weight. Scale bars: (A) 10 cm; (B) 10 cm. Source data are provided as a Source Data file.

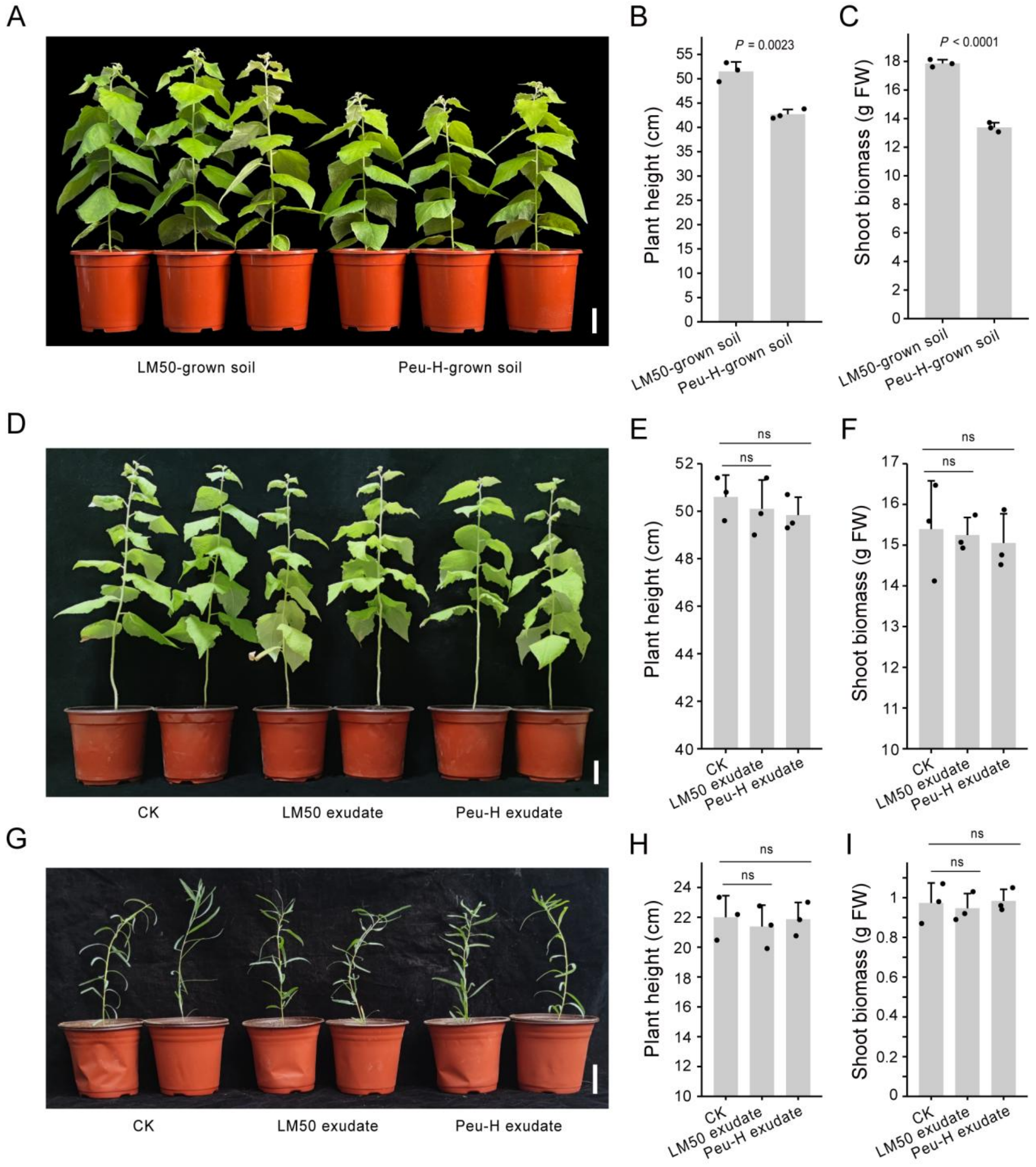

Supplementary Fig. 2 Root microbial community shaped by the vigorous genotype was more conducive to poplar growth. (A) Morphological differences of 84K transplants in different soils (LM50-grown soil or Peu-H-grown soil). Plant height **(B)** and fresh shoot biomass **(C)** of 84K transplants in different soils (LM50-grown soil or Peu-H-grown soil). *n* = 3 biologically independent samples. **(D)** Morphological differences of 84K after applying different poplar root exudates (LM50 exudate or Peu-H exudate). Plant height **(E)** and fresh shoot biomass **(F)** of 84K after applying different poplar root exudates (LM50 exudate or Peu-H exudate). *n* = 3 biologically independent samples. **(G)** Morphological differences of Peu-H after

applying different poplar root exudates (LM50 exudate or Peu-H exudate). Plant height **(H)** and fresh shoot biomass **(I)** of Peu-H after applying different poplar root exudates (LM50 exudate or Peu-H exudate). n = 3 biologically independent samples (two-sided Student's t-test, ns: not significant). Each bar represents the mean ± SEM. FW, fresh weight. Scale bars: (A) 5 cm; (D) 5 cm; (G) 5 cm. Source data are provided as a Source Data file.

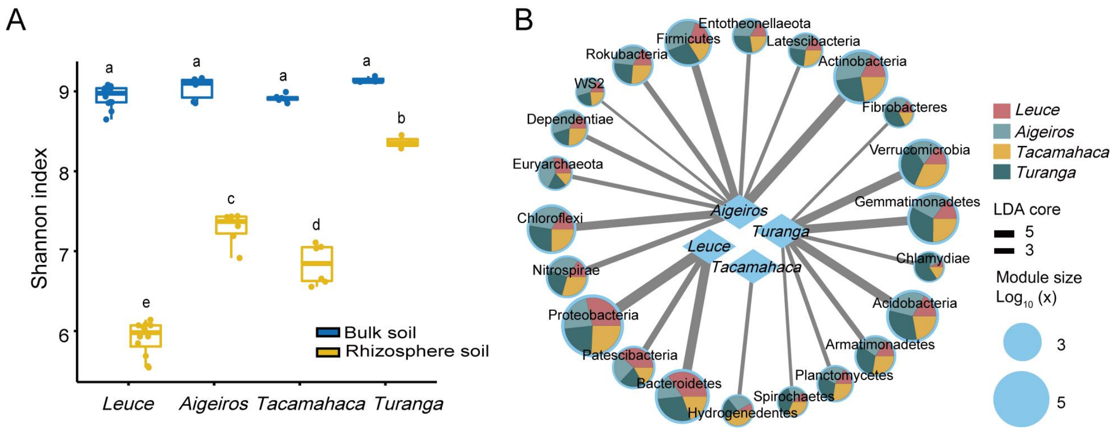

Supplementary Fig. 3 Distinction of root microbiomes in poplar. (A) Differences in shannon diversity between bulk soils and rhizosphere soils in four sections. n_{Leuce} = 12, $n_{Aigeiros}$ = 6, $n_{Tacamahaca}$ = 6, $n_{Turanga}$ = 3; biologically independent samples. The horizontal bars within the boxes represent medians. The tops and bottoms of the boxes represent the 75th and 25th percentiles, respectively. The upper and lower whiskers extend no further than 1.5 times the interquartile range from the upper edge or lower edge of the box. **(B)** Linear discriminant analysis effect size (LEfSe) was performed to identify the phylum-level rhizosphere bacteria that are differentially represented between the different poplar sections. Edge thickness denotes the LDA score (LDA score > 2, two-sided Kruskal-Wallis test, FDR adjusted *P*-values < 0.05). The size of the pie chart represents the abundance of microorganisms, and the color area of the pie chart indicates the distribution ratio of the microbe between different sections. Different letters indicate significantly different groups (One-way ANOVA, *P*-values < 0.05; *P*-values are shown in Source Data file). Source data are provided as a Source Data file.

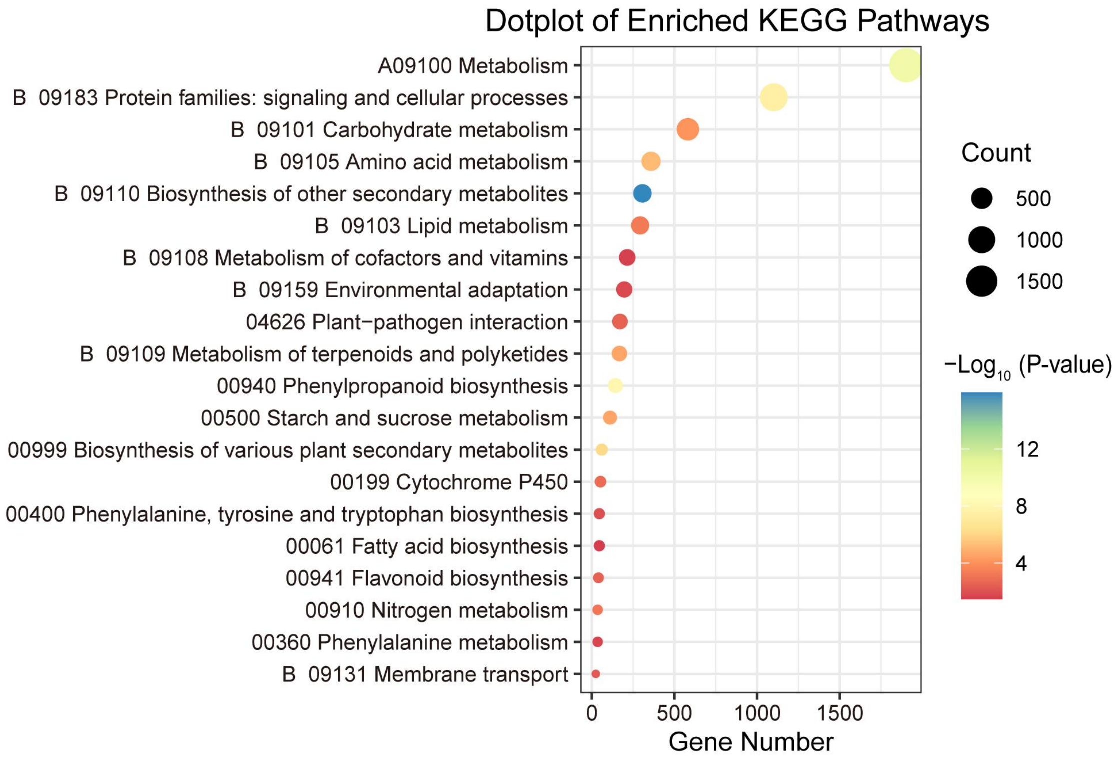

Supplementary Fig. 4 KEGG enrichment analyses of all differentially expressed genes. Only 20 significantly enriched functional modules were shown (One-sided Fisher exact-test, *P*-values < 0.05). Source data are provided as a Source Data file.

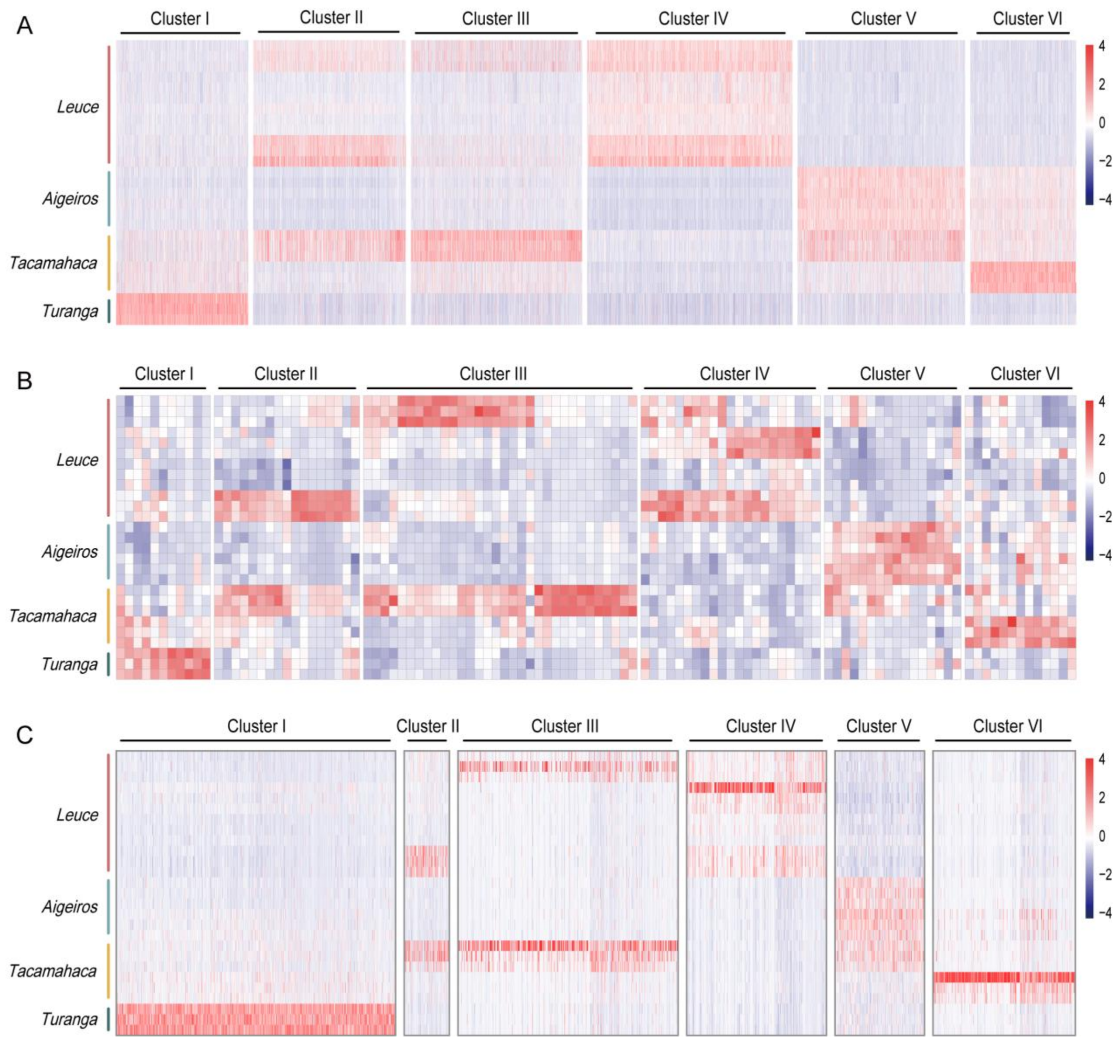

Supplementary Fig. 5 Gene expression, flavonoid accumulation, and microbe enrichment patterns in poplar roots. (A) Expression patterns of 17,698 genes co-expressed (two-sided; Pearson; $r \geq 0.7$, P-values < 0.01) with at least one flavonoid in six co-expression clusters. Samples corresponded to different colors (each color corresponded to one section). **(B)** Accumulation patterns of flavonoids in six co-expression clusters. According to the *k*-means clustering algorithm, 110 differential flavonoids in 27 samples were divided into six clusters with different accumulation patterns, and 27 samples corresponded to different colors (each color corresponds to one section). **(C)** Enrichment patterns of 2,579 ASVs co-expressed (two-sided; Pearson; $r \geq 0.7$, P-values < 0.01) with at least one flavonoid in six co-expression clusters. Samples corresponded to different colors (each color corresponds to one section). Expression data were Z-score standardized. Source data are provided as a Source Data file.

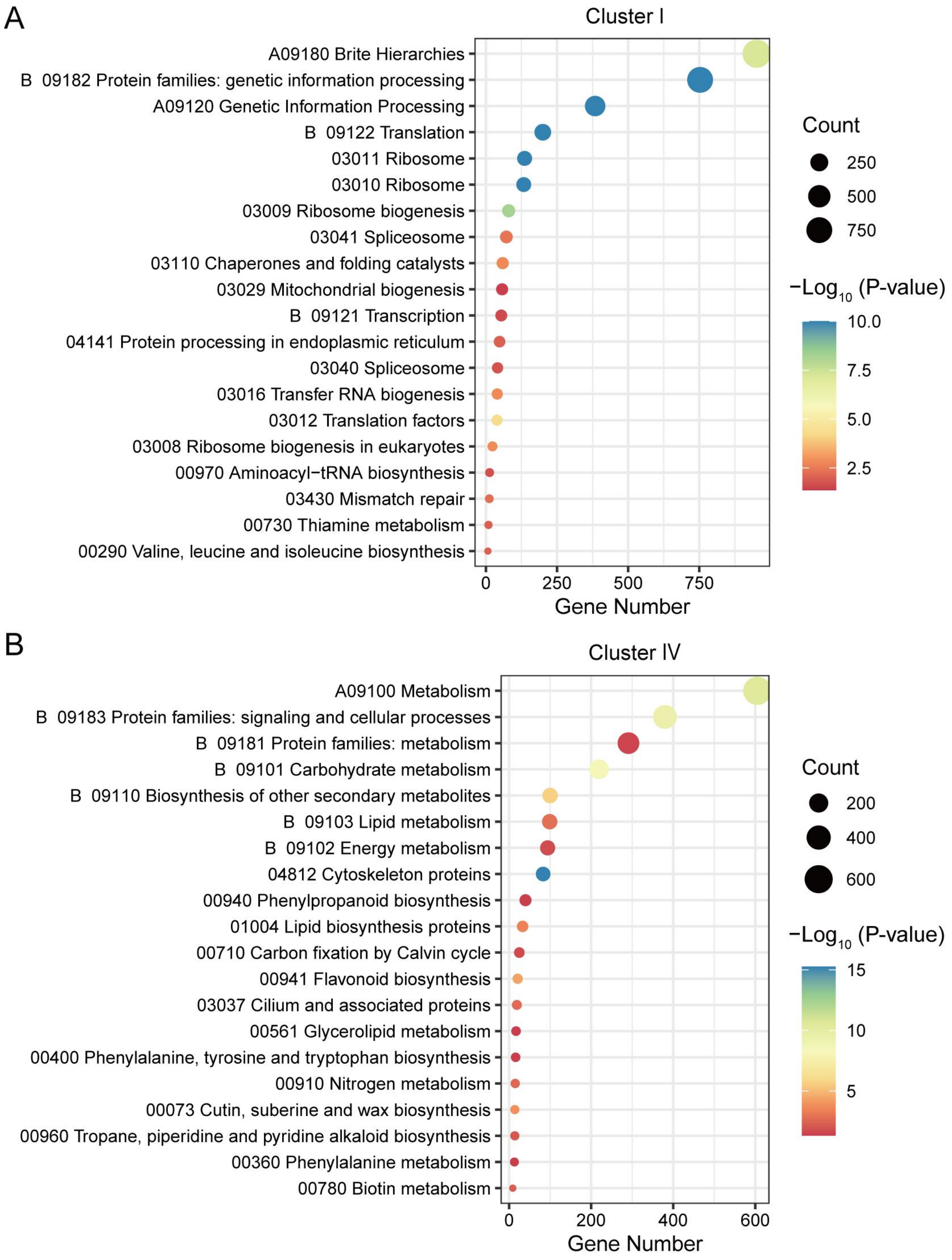

Supplementary Fig. 6 KEGG enrichment analyses of gene clusters. Functional enrichment analyses of gene Cluster I **(A)** and Cluster IV **(B)**. Only 20 significantly enriched functional modules were shown (One-sided Fisher exact-test, *P*-values < 0.05). Source data are provided as a Source Data file.

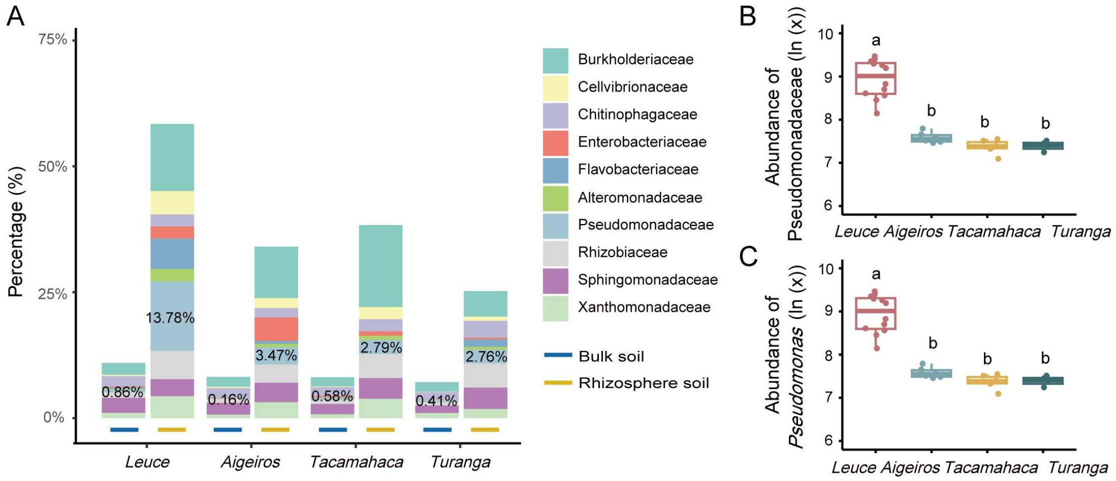

Supplementary Fig. 7 Relative abundance of Pseudomonadaceae and *Pseudomonas*. **(A)** Relative abundance of the top 10 rhizosphere microbial families in bulk soil and rhizosphere soil. The numbers are the relative abundances of Pseudomonadaceae. The abundance of Pseudomonadaceae **(B)** and *Pseudomonas* **(C)** in the rhizosphere of four sections. $n_{Leuce} = 12$, $n_{Aigeiros} = 6$, $n_{Tacamahaca} = 6$, $n_{Turanga} = 3$; biologically independent samples. The horizontal bars within the boxes represent medians. The tops and bottoms of the boxes represent the 75th and 25th percentiles, respectively. The upper and lower whiskers extend no further than 1.5 times the interquartile range from the upper edge or lower edge of the box. Different letters indicate significantly different groups (One-way ANOVA, P-values < 0.05; P-values are shown in Source Data file). Source data are provided as a Source Data file.

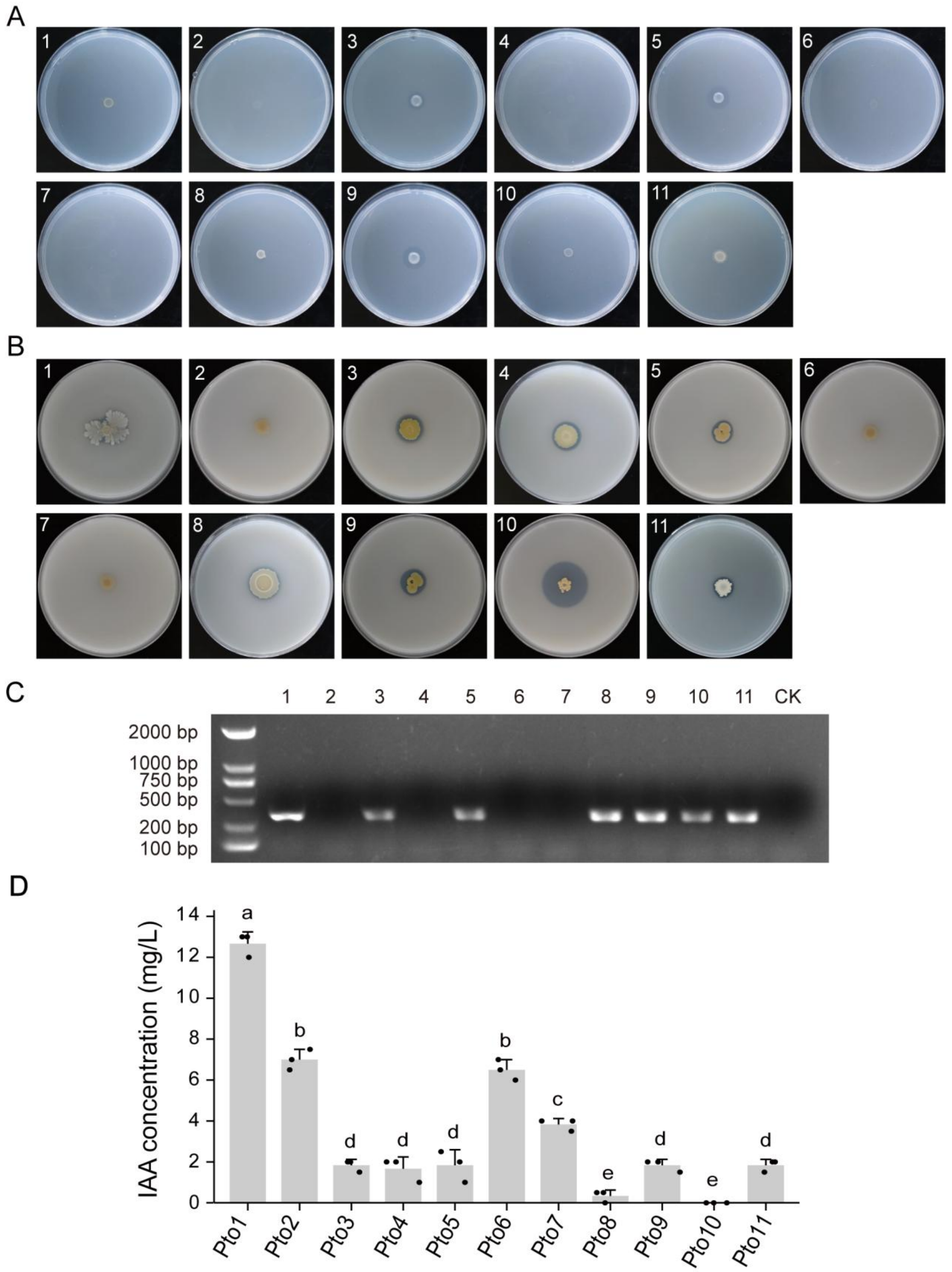

Supplementary Fig. 8 Determination of beneficial function of *Pseudomonas* strains. **(A)** Determination of growth ability of strains on nitrogen-fixing medium (Ashby nitrogen-free solid medium). **(B)** Determination of growth ability of strains on PKO inorganic phosphate solid medium. **(C)** The *nif*H genes were cloned by nested PCR. The target fragment length was approximately 360 bp. **(D)** The IAA production capacity of strains was detected in the presence of tryptophan. 1: Pto1; 2: Pto2; 3:

Pto3; 4: Pto4; 5: Pto5; 6: Pto6; 7: Pto7; 8: Pto8; 9: Pto9; 10: Pto10; 11: Pto11. n = 3 biologically independent samples. Different letters indicate significantly different groups (One-way ANOVA, P-values < 0.05; P-values are shown in Source Data file). Each bar represents the mean ± SEM. Source data are provided as a Source Data file.

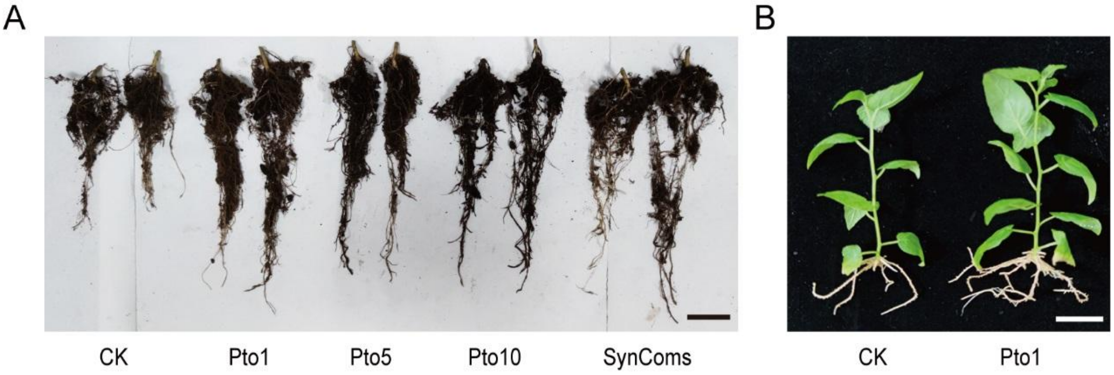

Supplementary Fig. 9 Effect of pseudomonad inoculation on fitness in poplar. (A) Poplar (84K) roots inoculated with pseudomonads in nitrogen-poor soil. **(B)** Growth differences of poplar (84K) inoculated with Pto1 in sterile nitrogen-rich medium. Scale bars: (A) 5 cm; (B) 2 cm.

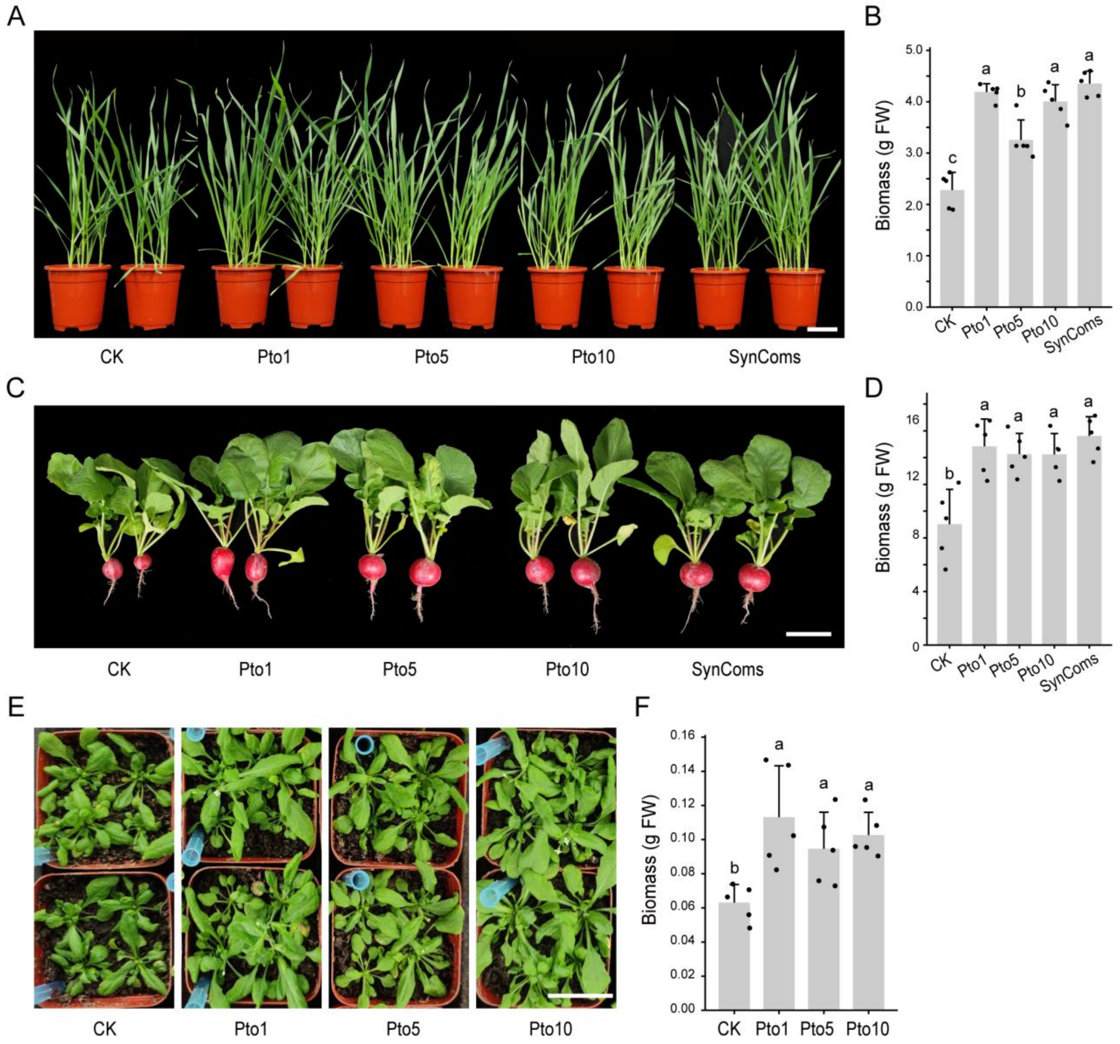

Supplementary Fig. 10 Effect of pseudomonad inoculation on fitness in other plants. Growth differences and total fresh biomass of wheat (*Triticum aestivum* L.; **A**, **B)**, radish (*Raphanus sativus* L.; **C**, **D)**, and Arabidopsis (*Arabidopsis thaliana* L.; **E**, **F)** inoculated with pseudomonads in nitrogen-poor soil. n = 5 biologically independent samples. Different letters indicate significantly different groups (One-way ANOVA, P-values < 0.05; P-values are shown in Source Data file). Each bar represents the mean ± SEM. FW, fresh weight. Scale bars: (A) 5 cm, (C) 5 cm, (E) 4 cm. Source data are provided as a Source Data file.

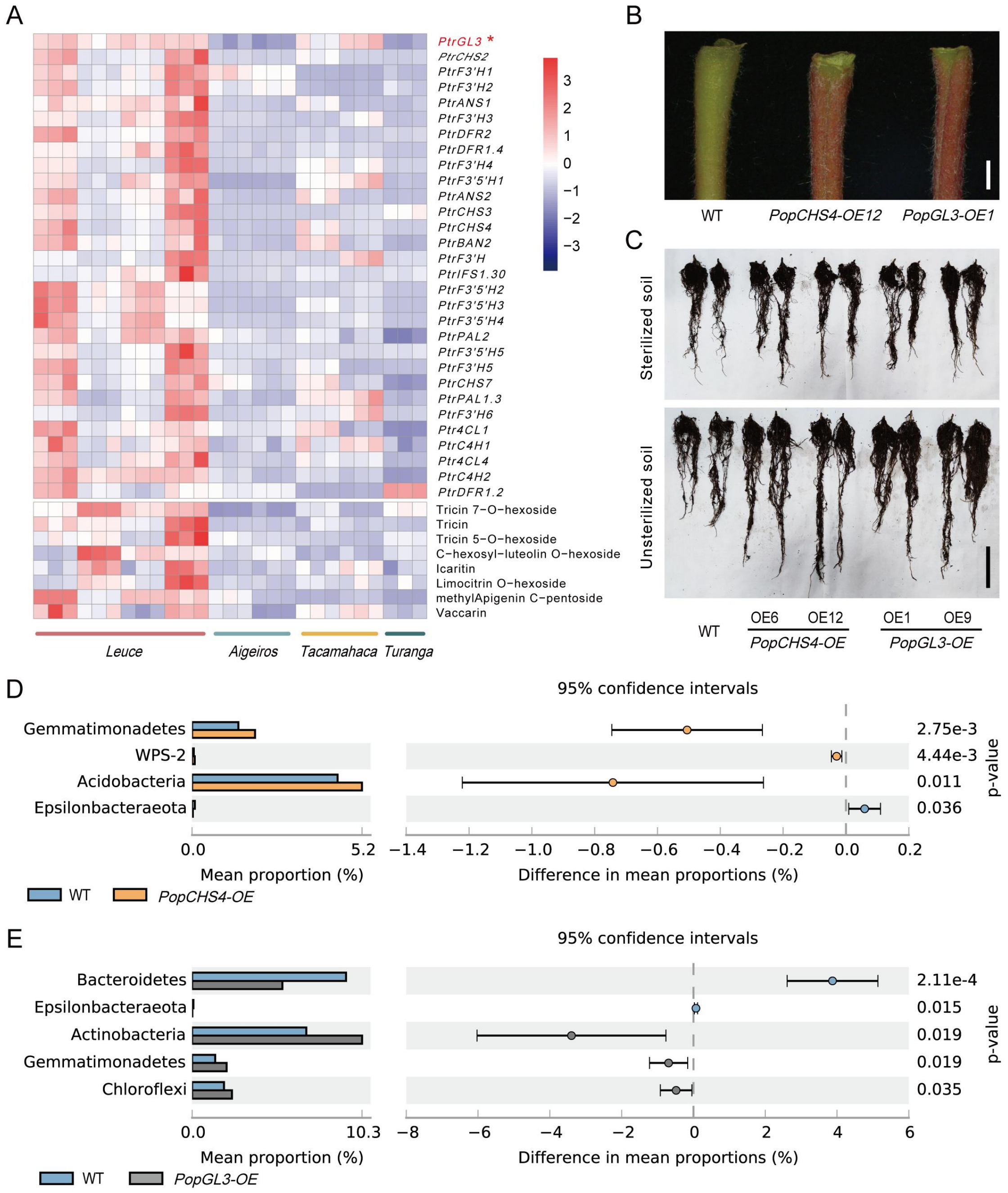

Supplementary Fig. 11 *PopGL3* regulates flavonoid synthesis to shape the rhizosphere microbial composition of poplar. (A) Heat map of *PopGL3* co-expression with flavonoid genes and flavones in root systems of different poplar sections. 27 samples correspond to different colors (each color corresponds to one section). The asterisk denotes that *GL3* is significantly enriched in *Leuce* (One-way ANOVA, *P*-values < 0.05). The leaf petioles **(B)** and roots **(C)** of the WT, *PopCHS4-OE*, and *PopGL3-OE* poplar lines. Abundance differences between *PopCHS4-OE* **(D)** and *PopGL3-OE* **(E)** poplar lines and WT rhizosphere

microbiomes at the phylum level (two-sided Welch's t-test, by STAMP). $n_{\mathrm{WT}} = 3$, $n_{PopCHS4\text{-}OE} = 6$, $n_{PopGL3\text{-}OE} = 6$; biologically independent samples. Scale bars: (B) 1000 μm, (C) 10 cm. Source data are provided as a Source Data file.

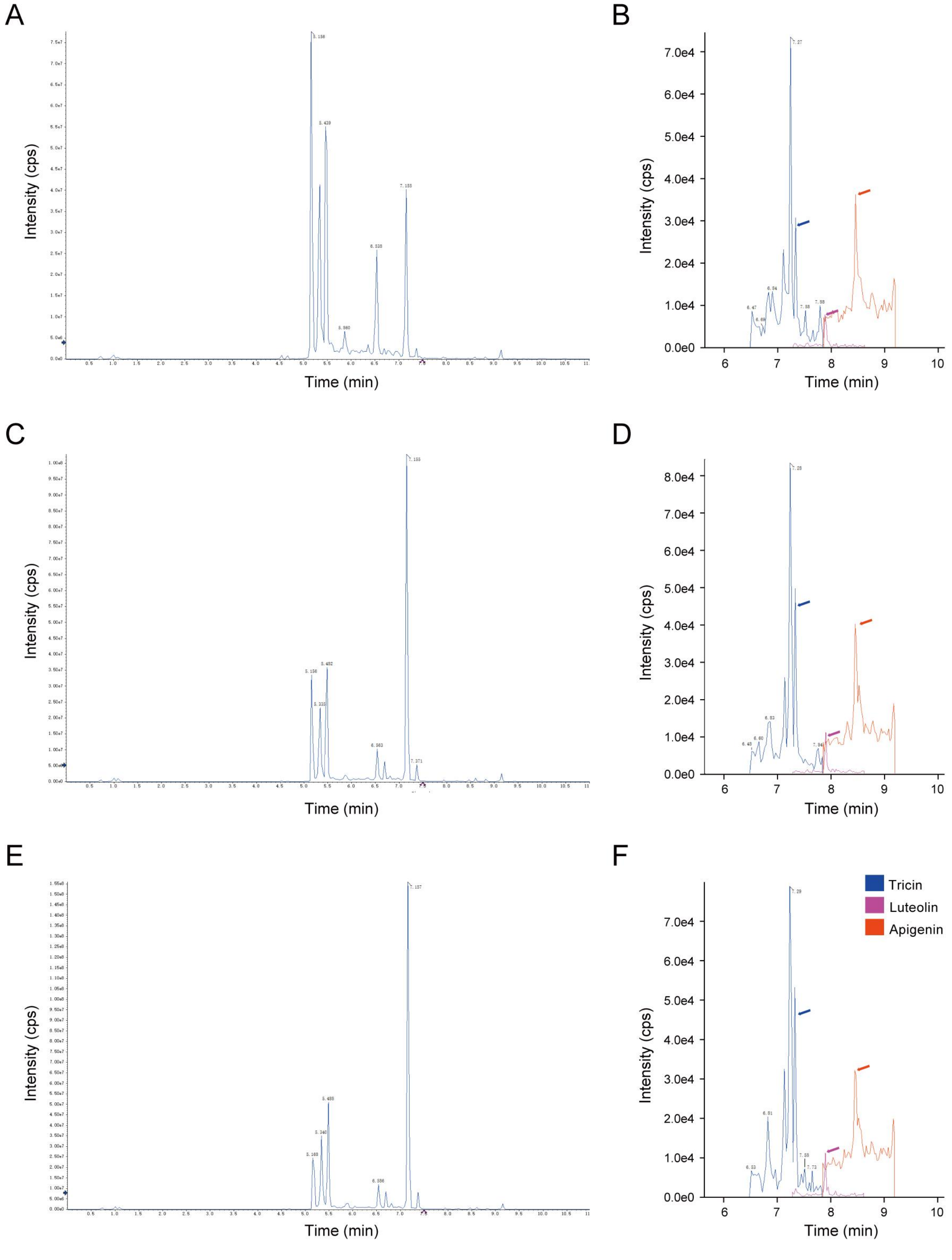

Supplementary Fig. 12 Detection and identification of flavonoid metabolites signals in the root by LC-MS/MS. The TIC (total ion chromatograms) for WT (**A**), *PopCHS4-OE12* (**C**), and *PopGL3-OE1* (**E**) poplar lines of roots. The XIC (Extracted ion chromatograms) for WT (**B**), *PopCHS4-OE12* (**D**), and *PopGL3-OE1* (**F**) poplar lines of roots.

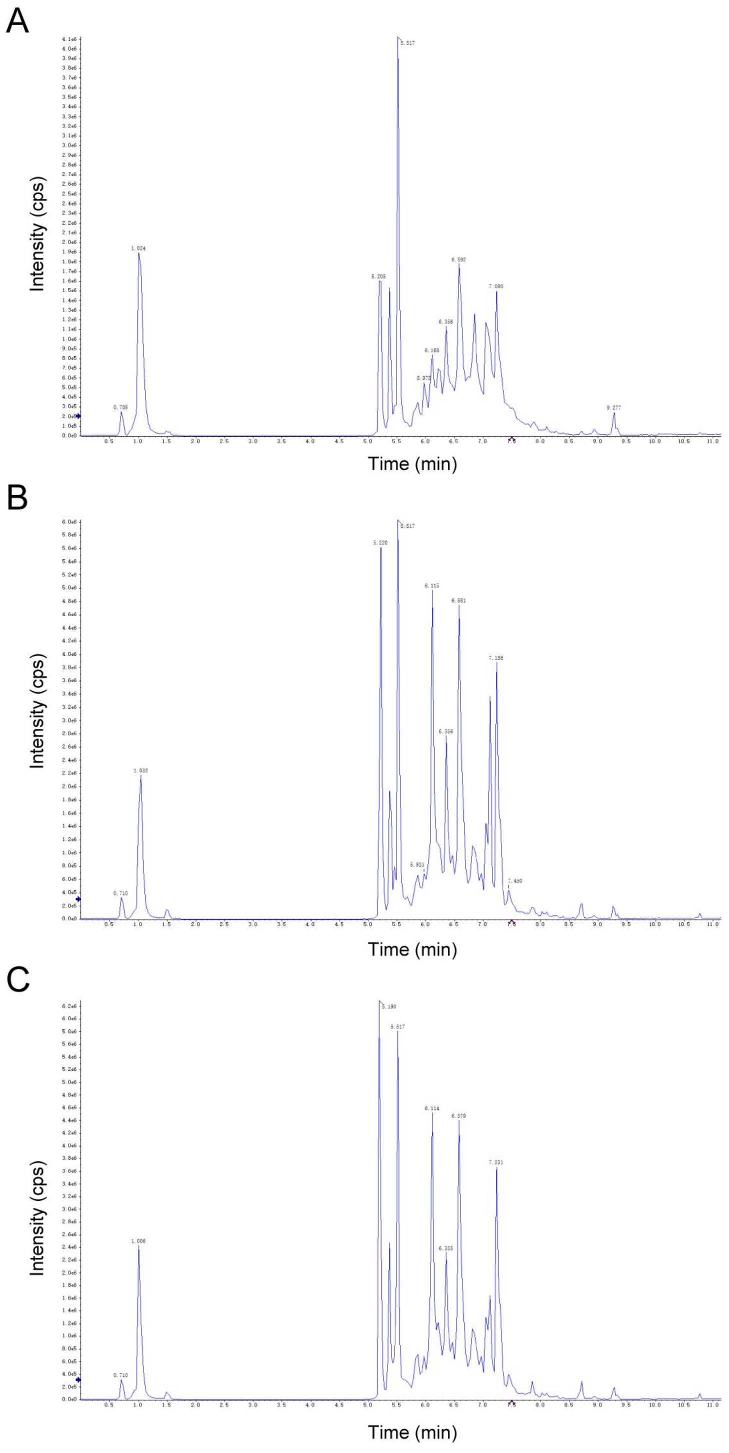

Supplementary Fig. 13 Detection and identification of flavonoid metabolites signals in the root exudate by LC-MS/MS. The TIC (total ion chromatograms) for WT (**A**), *PopCHS4-OE12* (**B**), and *PopGL3-OE1* (**C**) poplar lines of root exudates.

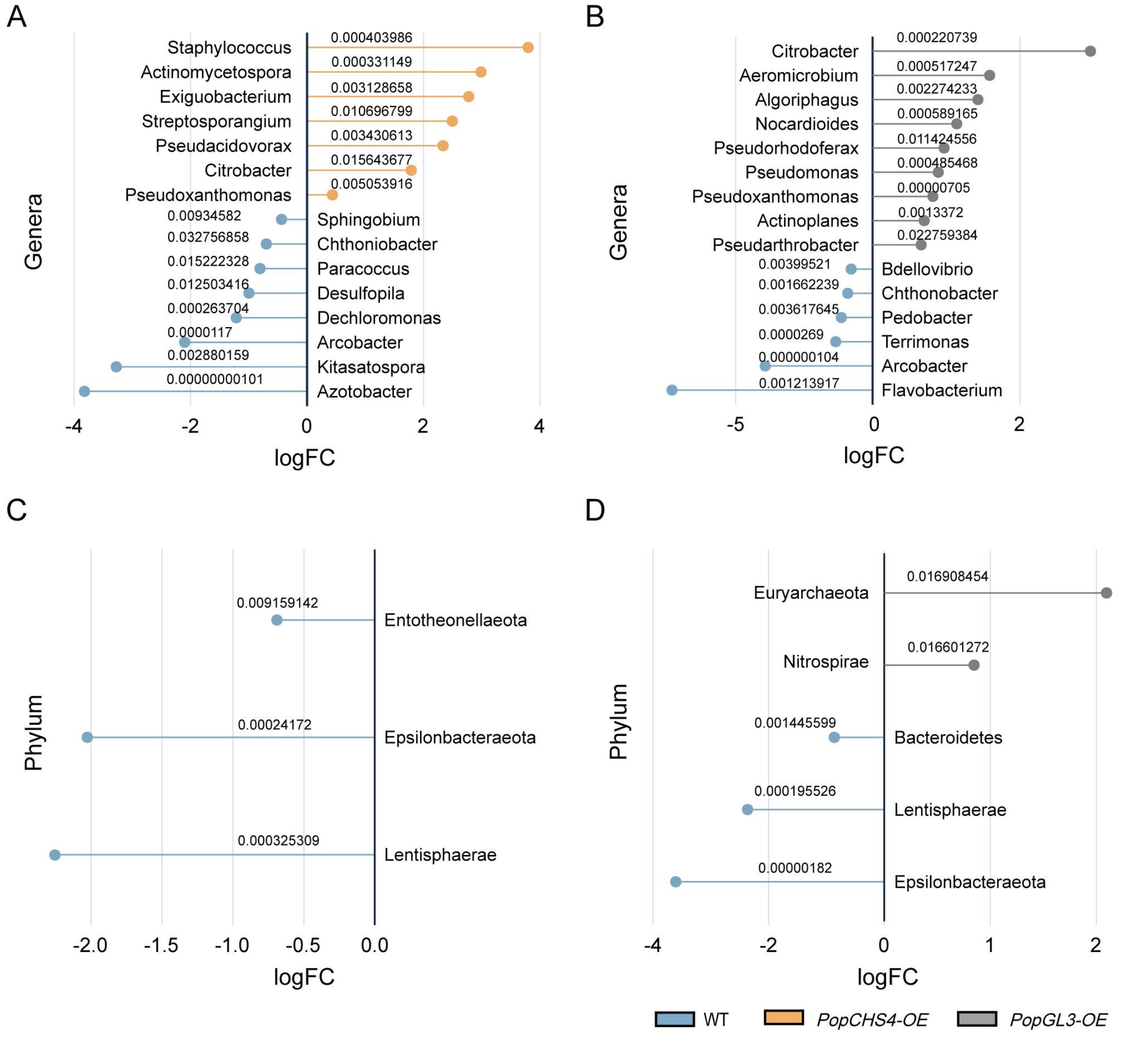

Supplementary Fig. 14 Abundance differences of rhizosphere microbiome between transgenic poplars and wild type. Compared with the wild types, the rhizosphere of *PopGL3-OE* poplar lines was significantly enriched with *Pseudomonas*, while *PopCHS4-OE* poplar lines were not. Abundance differences (*P*-values < 0.05; two-sided ANCOM-BC2) between *PopCHS4-OE* poplar lines and WT rhizosphere microbiomes at the phylum level **(A)** and genera level **(C)**. Abundance differences (*P*-values < 0.05; two-sided ANCOM-BC2) between *PopGL3-OE* poplar lines and WT rhizosphere microbiomes at the phylum level **(B)** and genera level **(D)**. $n_{WT} = 3$, $n_{PopCHS4\text{-}OE} = 6$, $n_{PopGL3\text{-}OE} = 6$; biologically independent samples. Source data are provided as a Source Data file.

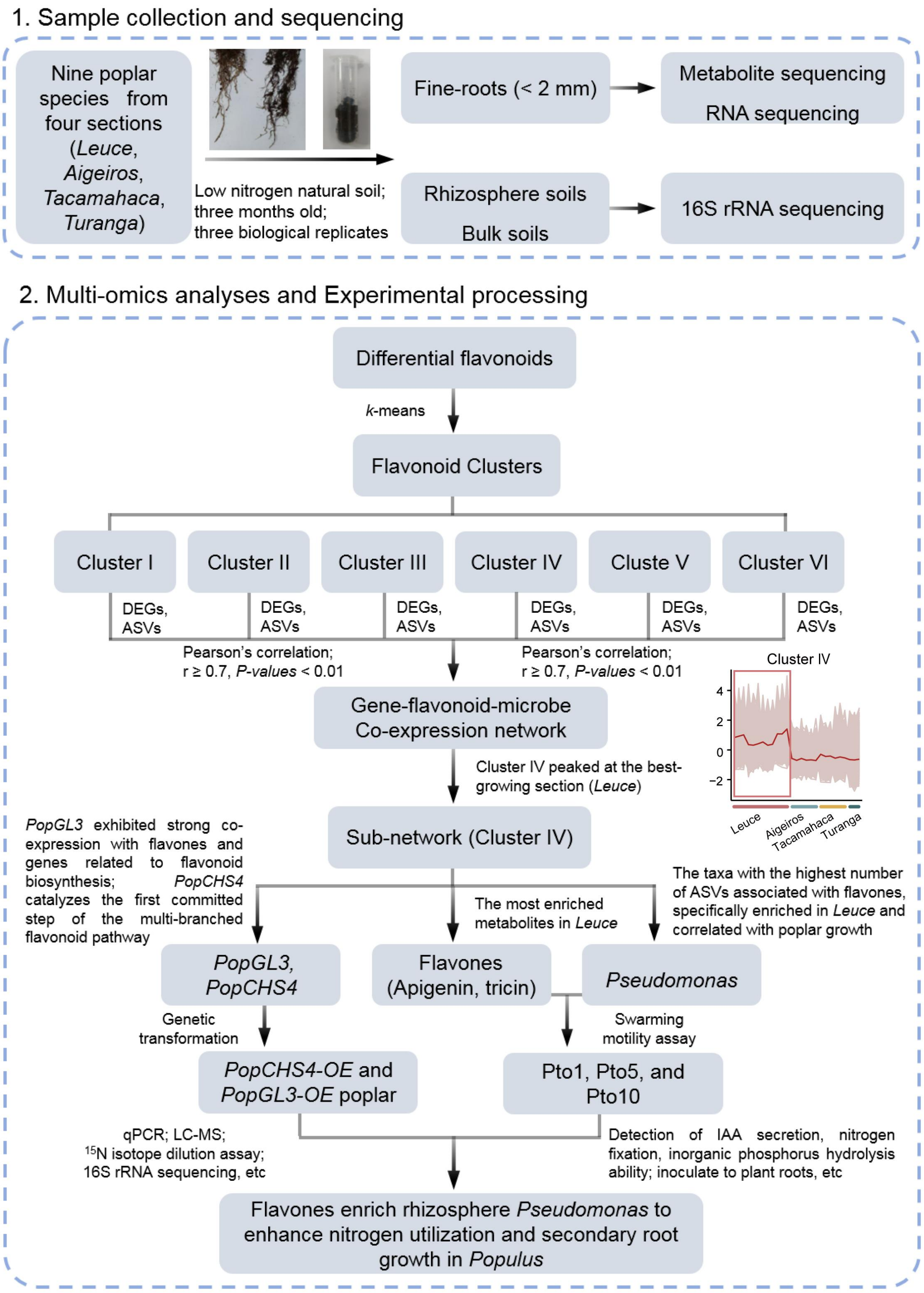

Supplementary Fig. 15 Conceptual diagram of experimental design of this study. 1. Sample collection and sequencing: Fine-root (< 2 mm), rhizosphere soil, and bulk soil samples were collected from nine poplar species across four sections (*Leuce*, *Aigeiros*, *Tacamahaca*, *and Turanga*) cultivated in low-nitrogen natural soil for three

months. Three biological replicates per species. Transcriptome and flavonoid metabolite sequencing were conducted on root samples; 16S rRNA amplicon sequencing was performed on rhizosphere soil and bulk soil samples. 2. Multi-omics analyses and Experimental processing: Multi-omics analyses were performed on all datasets to elucidate the intricate mechanisms of gene-metabolite-microbe interactions. Differential flavonoids were classified into six clusters based on their accumulation patterns using the *k*-means clustering algorithm, and a rigorous correction (two-sided; Pearson; $r \geq 0.7$, P-values < 0.01) was employed to screen for DEGs and ASVs that were significantly associated with the flavonoids in each cluster. Since Cluster IV peaked at the *Leuce*, which exhibited the best growth performance, the genes, metabolites, and microbes in Cluster IV were selected for detailed subnetwork analysis with poplar phenotypes. Within the metabolic cluster, flavones such as tricin and apigenin (with their derivatives) were the most abundant and significantly enriched in *Leuce*. Notably, Pseudomonadaceae represented the most numerous taxa in Cluster IV, with 62 ASVs (all ASVs belong to the *Pseudomonas*). Correlation analysis revealed that genes related to flavonoid biosynthesis and flavones exhibited the strongest association with Pseudomonadaceae and *Pseudomonas*, while the increased abundance of Pseudomonadaceae and *Pseudomonas* was highly correlated with the growth characteristics of poplar. Additionally, the transcription factor *PopGL3* was found to be co-expressed with flavones and flavonoid biosynthesis genes in Cluster IV. Consequently, *PopGL3*, flavones (apigenin, tricin), and *Pseudomonas* were selected for further investigation. *PopCHS4-OE* (*CHS* catalyzes the first committed step of the multi-branched flavonoid pathway) and *PopGL3-OE* lines were obtained via the 84K poplar genetic transformation method, and then qPCR, LC-MS, and amplicon sequencing were carried out on the poplar transgenic lines. Swarming motility assay indicated that apigenin and tricin significantly enhance the motility of pseudomonad isolates (Pto1, Pto5, and Pto10). Subsequently, Pto1, Pto5, and Pto10 were inoculated in poplar and other plant roots for growth promotion experiments. The methods or other conditions for each step are listed around the steps. For further details, please see the Methods of the manuscript.

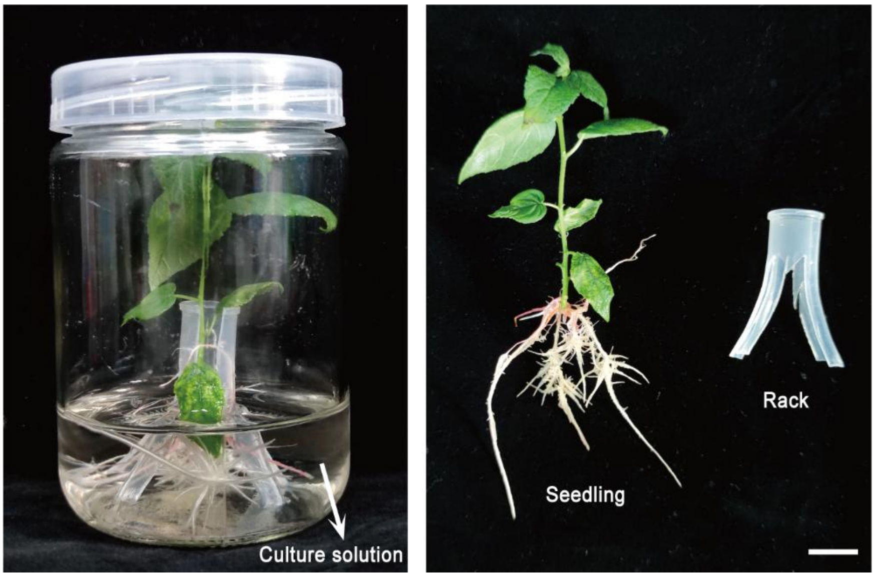

Supplementary Fig. 16 Sterile hydroponic culture apparatus of poplar. The sterile culture apparatus was used for the collection of root secretions under sterile conditions and the colonization experiments of RFP-tagged Pto1 in the root. Scale bar: 1 cm.

Supplementary Tables

Supplementary Table 1. Basic chemical properties of soils.

	SOM (g/kg)	TN (%)	AP (mg/kg)	AK (mg/kg)
LM50 bulk soil	23.6 b	0.070 b	11.9 a	139.1 b
Peu-H bulk soil	23.9 ab	0.072 b	12.2 a	142.7 b
LM50 rhizosphere soil	26.2 a	0.084 a	10.6 b	153.2 a
Peu-H rhizosphere soil	25.7 a	0.087 a	10.1 b	157.4 a

Note: TN: total nitrogen, SOM: soil organic matter, AP: available phosphorus, AK: available potassium. n = 3 biologically independent samples. Different letters indicate significantly different groups (One-way ANOVA, P-values < 0.05). SOM, df = 3, F = 6.17, P-values = 0.017796; TN, df = 3, F = 54.19, P-values = 0.000012; AP, df = 3, F = 13.32, P-values = 0.001773; AK, df = 3, F = 52.94, P-values = 0.000013.

Supplementary Table 2. Contribution of biological N-fixation by different strains or SynComs to N nutrition of the shoots of poplars grown in soil containing ^{15}N.

Teatment	Contain ^{15}N (%)	%Ndfa	N_2-fixed (mg)
CK	1.5784	--	--
Pop1	1.4171	10.21	5.01
Pop5	1.4792	6.28	2.83
Pop10	1.4934	5.39	2.55
Syncoms	1.3987	11.38	6.03

Note: Values represent the mean of three replicates.

Supplementary Table 3. Contribution of biological N-fixation by transgenic plant root microorganisms to N nutrition of the shoots of poplars grown in unsterilized soil containing ^{15}N.

Genetype	Contain ^{15}N (%)	%Ndfa	N_2-fixed (mg)
WT	1.8661	--	--
PopCHS4-OE6	1.7452	6.48	3.49
PopCHS4-OE12	1.7691	5.20	2.99
PopGL3-OE1	1.7256	7.53	4.15
PopGL3-OE9	1.7417	6.67	3.69

Note: Values represent the mean of three replicates.

Supplementary Table 4. Details of nine poplar species in four sections.

Sections	Species	Abbreviations	Sources
Leuce	*P. alba* × *P. glandulosa* Y	Pal-Y	Shandong, China
	P. tomentosa × *P. bolleana* M	Pto-M	Beijing, China
	P. alba × *P. glandulosa* 84K	84K	Korea
	P. tomentosa Lumao50	LM50	Shandong, China
Aigeiros	*P. euramericana* 74/76	107	Shandong, China
	P. euramericana H3-1	H3-1	Shandong, China
Tacamahaca	*P. szechuanica* Z	Psz-Z	Sichuan, China
	P. trichocarpa M	Pot-M	America
Turanga	*P. euphratica* H	Peu-H	Xinjiang, China

Supplementary Table 5. Details of four poplar forest soils.

Sections	Locations	Abbreviations	Sources
Leuce soil	E: 115°22'8", N: 36°30'54"	*Leuce*	Guanxian state-owned P. tomentosa forest farm
Aigeiros soil	E: 115°22'8", N: 36°30'54"	*Aigeiros*	Guanxian state-owned P. tomentosa forest farm
Tacamahaca soil	E: 115°22'8", N: 36°30'54"	*Tacamahaca*	Guanxian state-owned P. tomentosa forest farm
Turanga soil	E: 80°15'18", N: 40°45'39"	*Turanga*	Akesu Danglang tribe

References

1. Caporaso, J. G. et al. QIIME allows analysis of high-throughput community sequencing data. *Nat. Methods*. **7**, 335-336 (2010).
2. Edgar, R. C. Search and clustering orders of magnitude faster than BLAST. *Bioinformatics*. **26**, 2460-2461 (2010).
3. Andrews, S. FastQC: a quality control tool for high throughput sequence data. http://www.bioinformatics.babraham.ac.uk/projects/fastqc (2010).
4. Edgar, R. C. UNOISE2: improved error-correction for Illumina 16S and ITS amplicon sequencing. *Biorxiv*, 81257 (2016).
5. Quast, C. et al. The SILVA ribosomal RNA gene database project: improved data processing and web-based tools. *Nucleic. Acids. Res.* **41**, D590-D596 (2013).
6. Lu, J. & Salzberg, S. L. Ultrafast and accurate 16S rRNA microbial community analysis using Kraken 2. *Microbiome*. **8**, 124 (2020).
7. Lima, J. et al. Taxonomic annotation of 16S rRNA sequences of pig intestinal samples using MG-RAST and QIIME2 generated different microbiota compositions. *J. Microbiol. Methods*. **186**, 106235 (2021).
8. Segata, N. et al. Metagenomic biomarker discovery and explanation. *Genome Biol.* **12**, R60 (2011).
9. Parks, D. H., Tyson, G. W., Hugenholtz, P. & Beiko, R. G. STAMP: statistical analysis of taxonomic and functional profiles. *Bioinformatics*. **30**, 3123-3124 (2014).
10. Lin, H. & Peddada, S. D. Multigroup analysis of compositions of microbiomes with covariate adjustments and repeated measures. *Nat. Methods*. **21**, 83-91 (2024).
11. Li, Y. et al. MicroTom Metabolic Network: Rewiring Tomato Metabolic Regulatory Network throughout the Growth Cycle. *Mol. Plant.* **13**, 1203-1218 (2020).
12. Chen, S., Zhou, Y., Chen, Y. & Gu, J. fastp: an ultra-fast all-in-one FASTQ

preprocessor. *Bioinformatics*. **34**, i884-i890 (2018).

13. Kim, D., Paggi, J. M., Park, C., Bennett, C. & Salzberg, S. L. Graph-based genome alignment and genotyping with HISAT2 and HISAT-genotype. *Nat. Biotechnol.* **37**, 907-915 (2019).
14. Pertea, M. et al. StringTie enables improved reconstruction of a transcriptome from RNA-seq reads. *Nat. Biotechnol.* **33**, 290-295 (2015).
15. Bu, D. et al. KOBAS-i: intelligent prioritization and exploratory visualization of biological functions for gene enrichment analysis. *Nucleic. Acids. Res.* **49**, W317-W325 (2021).
16. Wen, B., Mei, Z., Zeng, C. & Liu, S. metaX: a flexible and comprehensive software for processing metabolomics data. *Bmc Bioinformatics*. **18**, 183 (2017).
17. Glickmann, E. & Dessaux, Y. A critical examination of the specificity of the salkowski reagent for indolic compounds produced by phytopathogenic bacteria. *Appl. Environ. Microbiol.* **61**, 793-796 (1995).
18. Montañez, A., Abreu, C., Gill, P. R., Hardarson, G. & Sicardi, M. Biological nitrogen fixation in maize (*Zea mays* L.) by ^{15}N isotope-dilution and identification of associated culturable diazotrophs. *Biol. Fertil. Soils*. **45**, 253-263 (2009).
19. Bartlett, A. et al. Mapping genome-wide transcription-factor binding sites using DAP-seq. *Nat. Protoc.* **12**, 1659-1672 (2017).
