# Supplementary material for: Flavones enrich rhizosphere Pseudomonas to enhance nitrogen utilization and secondary root growth in Populus
Source: Nat Commun. 2025 Feb 7;16:1461. doi: 10.1038/s41467-025-56226-w (PMC11805958; doi:10.1038/s41467-025-56226-w)
Supplement: Supplementary file 3 — Description of Additional Supplementary Files [file 41467_2025_56226_MOESM3_ESM.pdf]

Description of Additional Supplementary Files

Supplementary Data Legends:

Supplementary Data 1. The eleven growth phenotypes of nine poplar species.

Supplementary Data 2. The specific marker taxa analysis of rhizosphere microorganisms in different sections.

Supplementary Data 3. Genus-level relative abundance (mean) of rhizosphere microorganisms in four poplar sections.

Supplementary Data 4. The Permutational Multivariate Analysis of Variance (PERMANOVA) analyses of datasets.

Supplementary Data 5. The differentially expressed genes detected between the four sections.

Supplementary Data 6. The KEGG enrichment analyses of differentially expressed genes and co-expression gene clusters.

Supplementary Data 7. The differentially produced root metabolites at least within one section versus anothor.

Supplementary Data 8. The co-response clusters based on the transcriptome, metabolome, and bacterial community data.

Supplementary Data 9. The flavonoid biosynthesis related enzyme genes of coexpression gene clusters.

Supplementary Data 10. Sequence (16S rRNA) of the Pseudomonas isolates.

Supplementary Data 11. Difference analysis of rhizosphere microbial abundance between transgenic poplars (PopCHS4-OE and PopGL3-OE) and wild type (WT; by ANCOM-BC2).

Supplementary Data 12. The primers used in this study.

Supplementary Data 13. The target gene sequences for this study.
