# Supplementary material for: Flavones enrich rhizosphere Pseudomonas to enhance nitrogen utilization and secondary root growth in Populus
Source: Nat Commun. 2025 Feb 7;16:1461. doi: 10.1038/s41467-025-56226-w (PMC11805958; doi:10.1038/s41467-025-56226-w)
Supplement: Supplementary file 17 — Reporting Summary [file 41467_2025_56226_MOESM17_ESM.pdf]

nature portfolio

Corresponding author(s): Jianbo Xie & Deqiang Zhang

Last updated by author(s): Dec 23, 2024

Reporting Summary

Nature Portfolio wishes to improve the reproducibility of the work that we publish. This form provides structure for consistency and transparency in reporting. For further information on Nature Portfolio policies, see our Editorial Policies and the Editorial Policy Checklist.

Statistics

For all statistical analyses, confirm that the following items are present in the figure legend, table legend, main text, or Methods section.

n/a	Confirmed	
☐	☒	The exact sample size (*n*) for each experimental group/condition, given as a discrete number and unit of measurement
☐	☒	A statement on whether measurements were taken from distinct samples or whether the same sample was measured repeatedly
☐	☒	The statistical test(s) used AND whether they are one- or two-sided<br>*Only common tests should be described solely by name; describe more complex techniques in the Methods section.*
☒	☐	A description of all covariates tested
☐	☒	A description of any assumptions or corrections, such as tests of normality and adjustment for multiple comparisons
☐	☒	A full description of the statistical parameters including central tendency (e.g. means) or other basic estimates (e.g. regression coefficient) AND variation (e.g. standard deviation) or associated estimates of uncertainty (e.g. confidence intervals)
☐	☒	For null hypothesis testing, the test statistic (e.g. *F*, *t*, *r*) with confidence intervals, effect sizes, degrees of freedom and *P* value noted<br>*Give P values as exact values whenever suitable.*
☒	☐	For Bayesian analysis, information on the choice of priors and Markov chain Monte Carlo settings
☒	☐	For hierarchical and complex designs, identification of the appropriate level for tests and full reporting of outcomes
☐	☒	Estimates of effect sizes (e.g. Cohen's *d*, Pearson's *r*), indicating how they were calculated

Our web collection on statistics for biologists contains articles on many of the points above.

Software and code

Policy information about availability of computer code

Data collection	Data collection is not applicable in this work. We generated new data by ourselves in this manuscript.
Data analysis	Transcriptome sequencing data analysis: fastp (v0.14.0), HISAT2 (v2-2.1.0), SAMtools (v0.1.19), StringTie (v1.3.6), KOBAS (v3.0) and DESeq2 (v1.34.0); 16S rRNA data processing: mothur (v1.39.5), QIIME2, FAPROTAX (v1.1) and STAMP (v2.1.3); Metabolites data: SCIEX OS (v1.4) and metaX (v2.71); Co-expression network by R (v4.1.3) package cluster (v2.1.4), WGCNA package (v1.71) and ggplot2 package (v3.3.6); Correlation analysis of modules: vegan package (v2.6-2) and Cytoscape (v3.9.1); DNA affinity purification sequencing (DAP-seq) and data analysis: Bowtie2 (v2.3.5), SAMtools (v0.1.19), MACS2 (v2.0), BEDtools (v2.25.0), ChIPseeker (v1.22.1) and Homer (v4.11); Leaf area calculation: ImageJ (v1.53q); Statistical analyses by SPSS (v25.0).

For manuscripts utilizing custom algorithms or software that are central to the research but not yet described in published literature, software must be made available to editors and reviewers. We strongly encourage code deposition in a community repository (e.g. GitHub). See the Nature Portfolio guidelines for submitting code & software for further information.

Data

Policy information about availability of data

All manuscripts must include a data availability statement. This statement should provide the following information, where applicable:

- Accession codes, unique identifiers, or web links for publicly available datasets
- A description of any restrictions on data availability
- For clinical datasets or third party data, please ensure that the statement adheres to our policy

The raw amplicon data are publicly accessible in the Genome Sequence Archive of the Beijing Institute of Genomics BIG Data Center, Chinese Academy of Sciences, under CRA015093 (nine poplar genetypes) and CRA015469 (PopCHS4-OE and PopGL3-OE poplar transgenic lines). The root transcriptome could be accessed under CRA015096. The DAPseq data could be accessed under CRA015475. The metabonomics raw data and other research data are available in the figshare database (10.6084/m9.figshare.26426578).

Research involving human participants, their data, or biological material

Policy information about studies with human participants or human data. See also policy information about sex, gender (identity/presentation), and sexual orientation and race, ethnicity and racism.

Reporting on sex and gender	*Use the terms sex (biological attribute) and gender (shaped by social and cultural circumstances) carefully in order to avoid confusing both terms. Indicate if findings apply to only one sex or gender; describe whether sex and gender were considered in study design; whether sex and/or gender was determined based on self-reporting or assigned and methods used. Provide in the source data disaggregated sex and gender data, where this information has been collected, and if consent has been obtained for sharing of individual-level data; provide overall numbers in this Reporting Summary. Please state if this information has not been collected. Report sex- and gender-based analyses where performed, justify reasons for lack of sex- and gender-based analysis.*
Reporting on race, ethnicity, or other socially relevant groupings	*Please specify the socially constructed or socially relevant categorization variable(s) used in your manuscript and explain why they were used. Please note that such variables should not be used as proxies for other socially constructed/relevant variables (for example, race or ethnicity should not be used as a proxy for socioeconomic status). Provide clear definitions of the relevant terms used, how they were provided (by the participants/respondents, the researchers, or third parties), and the method(s) used to classify people into the different categories (e.g. self-report, census or administrative data, social media data, etc.) Please provide details about how you controlled for confounding variables in your analyses.*
Population characteristics	*Describe the covariate-relevant population characteristics of the human research participants (e.g. age, genotypic information, past and current diagnosis and treatment categories). If you filled out the behavioural & social sciences study design questions and have nothing to add here, write "See above."*
Recruitment	*Describe how participants were recruited. Outline any potential self-selection bias or other biases that may be present and how these are likely to impact results.*
Ethics oversight	*Identify the organization(s) that approved the study protocol.*

Note that full information on the approval of the study protocol must also be provided in the manuscript.

Field-specific reporting

Please select the one below that is the best fit for your research. If you are not sure, read the appropriate sections before making your selection.

☒ Life sciences ☐ Behavioural & social sciences ☐ Ecological, evolutionary & environmental sciences

For a reference copy of the document with all sections, see nature.com/documents/nr-reporting-summary-flat.pdf

Life sciences study design

All studies must disclose on these points even when the disclosure is negative.

Sample size	No specific statistical methods were used to determine the sample size for the experiments. The sample size for each experiment was described completely in the Figure and Table legends or in Methods.
Data exclusions	There were no data points excluded from analysis in any of the experiments presented in this paper.
Replication	The root transcriptome, root metabolomics and microbiome experiment was performed once with three independent biological replicates (two individual Peu-H plants were pooled into one biological replicate). The validation experiments such as pseudomonads swarming motility and bacterial inoculation experiments were replicated at least twice. All attempts at replication were successful.
Randomization	In the same experiment, all plant materials were exposed to the same growth conditions. Plants of different genotypes or treatments were randomly placed.

Blinding

The root and soil samples for all nine genotypes were harvested blindly without access to genotype identities. Data analysis was blinded to genotypes and the researchers were allowed to choose each subject blindly.

Reporting for specific materials, systems and methods

We require information from authors about some types of materials, experimental systems and methods used in many studies. Here, indicate whether each material, system or method listed is relevant to your study. If you are not sure if a list item applies to your research, read the appropriate section before selecting a response.

Materials & experimental systems

n/a	Involved in the study	
☒	☐	Antibodies
☒	☐	Eukaryotic cell lines
☒	☐	Palaeontology and archaeology
☒	☐	Animals and other organisms
☒	☐	Clinical data
☒	☐	Dual use research of concern
☐	☒	Plants

Methods

n/a	Involved in the study	
☒	☐	ChIP-seq
☒	☐	Flow cytometry
☒	☐	MRI-based neuroimaging

Plants

Seed stocks

Tissue culture plantlets of poplar clones (84K, Pse-Z, Ptr-M, and Peu-H) were maintained in our laboratory, while the remaining five species (Pto-M, LM50, H3-1, Psz-Z and 107) were collected from the GuanXian state-owned P. tomentosa forest farm in Shandong Province, China (E: 115°22'8", N: 36°30'54") in April 2020. Materials for each genotype were collected from the same clone and sterile monoclonal tissue culture seedlings were obtained. Arabidopsis mutants plt3plt5plt7 and wox11wox12 were shared by Huchen Li and Viola Willemsen.

Novel plant genotypes

PopCHS4-OE and PopGL3-OE overexpressing lines were obtained by the Agrobacterium tumefaciens strain GV3101 mediated callus transformation method using P. alba × P. glandulosa 84K as material. Transgenic plants are propagated by clones, with two representative independent lines for each gene analysis and three biological replicates for each line.

Authentication

In this study, PCR identification was performed using two primers to determine whether the target genes were inserted into the 84K genome. The first set of primers were sequences at both ends of the polyclonal site inserted by the target gene. To eliminate GV3101 contamination, a second set of primers was designed based on the GV3101 virulence gene VirD2 sequence. The first group of primers has DNA bands, and the second group of primers without bands are transgenic lines.
